# Widening participation in medicine: what, why and how?

**DOI:** 10.15694/mep.2017.000184

**Published:** 2017-10-13

**Authors:** Rosemarie Patterson, Jim Price

**Affiliations:** 1 Brighton and Sussex Medical School

**Keywords:** Widening participation, Widening access, Access to medicine, Workforce mobility

## Abstract

This article was migrated. The article was marked as recommended.

Widening Participation (WP) is ‘the process of encouraging underrepresented socioeconomic groups to apply for Higher Education’. This is particularly relevant to medicine, where representation of lower socioeconomic groups is generally poor. Reducing this divide is necessary as socioeconomic diversity enhances social mobility and is likely to improve patient outcomes.

This review aims to explore the background to WP, including relevant political theory and also highlights the key methods currently used by medical schools to promote WP. These include pre-application measures (outreach and access to medicine courses), application interventions (contextual data, multiple-mini interviews and situational judgement tests) and post-application measures (foundation courses and ongoing support during medical school). The review also discusses the main criticisms of WP. Finally, it offers recommendations for medical schools regarding the implementation of WP initiatives.

## Background

Widening Participation (WP) is a term commonly used in the medical education literature. It has many synonyms (e.g. ‘widening access’) and understandings, however may be best defined as ‘ *the process of encouraging underrepresented socioeconomic groups to apply for Higher Education (HE)*’. ( Angel & Johnson, 2000; Cleland et al., 2014).

WP has specific relevance to medical education, where representation of lower socioeconomic groups is particularly poor ( BMA, 2009; Seyan et al., 2004). Medicine is historically an elitist course, studied only by those who could afford it - the white, male middle-class ( BMA, 2004; Townsend, 1968). This demonstrates a lack of diversity; defined by the BMA as “recognising that we are all different, and celebrating and valuing these differences”. This philosophy refers to the possibility for all individuals to have equal chances regardless of factors including (but not limited to) gender, ethnicity and age ( BMA, 2016; Young et al., 2012).

Medical school cohorts are now far more diverse in these areas, for example in 2015, 55% of students in medical school were female ( BMA, 2009; GMC, 2016; Milburn, 2012). When considering socioeconomic status (SES) however, medical schools are failing to achieve diversity ( Seyan et al., 2004). In 2008, 71% of successful medical school applicants came from socioeconomic classes one to three, whilst only 2% come from class seven (the lowest category) ( BMA, 2009). Lower SES students are therefore significantly less likely to both apply to medical school and have successful applications ( Brown & Garlick, 2006).

Generally, pupils of lower SES come from backgrounds where there is not a culture of HE. They may not have been encouraged to focus on their education, a factor directly associated with lower aspirations, or to undertake extra-curricular activities and relevant work experience ( Hill et al., 2004). Such pupils also tend to come from schools where high academic achievement is the exception: they therefore lack the advice of teachers experienced in helping students apply to medical school. Students are also influenced by peer relationships, establishing a social identity where they feel unable to access HE ( Maras, 2007). A final barrier preventing students accessing HE is finance, as the lowest socioeconomic backgrounds are the most ‘debt averse’ ( Callender & Jackson, 2005; Dismore, 2009; Hilton & Lewis, 2004).

Tackling these barriers to increase representation of lower SES groups in the medical profession is a necessity, broadly divided into two categories: social mobility and the improvement of healthcare provision.

Social mobility considers the ethical component of WP in medicine and refers to the ability of disadvantaged individuals to ‘move up in the world’ and vice versa ( Crawford et al., 2011). It focuses on equal opportunities, allowing all aspiring doctors the same chance of success in a medicine application, regardless of background. This is reflected by guidance from the British Medical Association, who highlight that disadvantaged students should be supported throughout medical education, to provide an equitable chance of vocational success ( BMA, 2009).

Increasing diversity (including through WP) amongst doctors is also likely to improve healthcare provision. The Medical Schools Council recommends that medical school graduates should have “social, cultural and ethnic backgrounds.. [which] reflect broadly the diversity of those they are called upon to serve” ( Angel & Johnson, 2000; McManus et al., 1998). This is particularly important to socioeconomic diversity, as lower socioeconomic groups over-proportionally access health services, due to factors such as poverty, poor housing and diet ( Angel & Johnson, 2000; Bedi & Gilthorpe, 2000). Increasing socioeconomic diversity within medicine may result in ‘like-treating-like’, with medical students from lower socioeconomic backgrounds being more likely to work in similar areas following graduation ( Brown & Garlick, 2006; James et a., 2008; Magnus & Mick, 2000). Access to a greater diversity of doctors may also support patients who feel isolated from the National Health Service due to cultural or similar barriers ( Cleland et al., 2014; Girotti et al., 2015).

Issues of WP have been recognised in UK policy since the election of the ‘New’ Labour Government and release of the Dearing Report in 1997 ( Pennell & West, 2005). This occurred in spite of recognition in the 1970s that knowledge distributed throughout society was necessary to support the economy and social mobility ( Hayton & Paczuska, 2003; Stevenson et al., 2010).

The Dearing Report recognised socioeconomic inequality in HE and highlighted the need for institutions to implement WP strategies ( Jones & Thomas, 2005). It also noted that the HE sector was well placed to address the imbalances, despite not being responsible for the underlying social inequality ( Lewis, 2002). In addition, it dictated the need for allocating appropriate funding to WP initiatives in HE institutions ( Chilosi et al., 2010). The Labour Government further recognised WP through their commitment for 50% of 18-30 year olds to have a HE qualification by 2010 ( Lewis, 2002), something that could only be achieved by increasing participation from disadvantaged groups ( Clarke, 2003; Taylor et al., 2009).

In response, the Higher Education Funding Council for England (HEFCE) undertook various measures to encourage WP in HE. They provided funding supplements to institutions enrolling disadvantaged students, incentivising them to generate WP aims and strategies, as well as special WP projects ( Lewis, 2002).

Despite these measures, the Higher Education Act (2004) marked the introduction of student loans and variable top-up fees, leading to concerns over socioeconomic equality in HE ( Kettley, 2007). To alleviate this, funding in WP was increased, with universities receiving resources to run outreach activities.

HEFCE now allocate funding to institutions supporting WP students through their university ‘life-cycle’. They also support HE networks for schools and oversee ‘Office for Fair Access’ agreements ( OFFA, 2015). These were introduced when the Coalition Government extended tuition fees to a maximum of £9000 per annum and required institutions charging over £6000 to allocate a proportion to WP measures. The level recommended was 15-30% of the tuition fee, depending on the institution ( OFFA, 2013).

## Widening participation strategies

**Figure 1.**
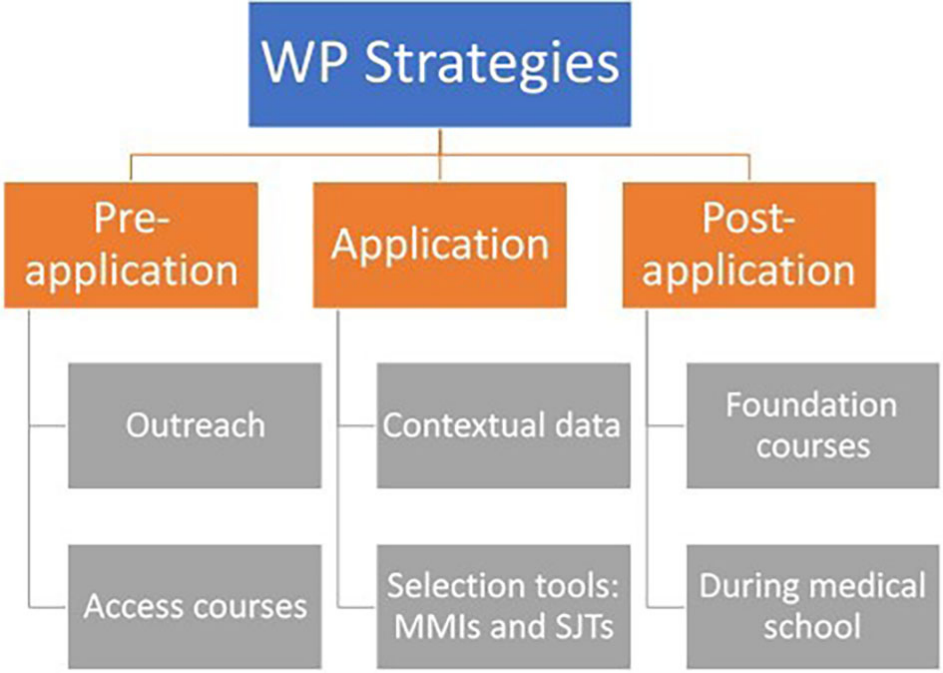
Details the various strategies used by medical schools to WP, created by the authors

There are various strategies for WP in medicine. These are commonly aimed at school students and application processes. Recently there has been consideration given to supporting lower SES students once they have reached medical school, to decrease attrition ( Figure 1).

### Pre-Application

Many individuals do not consider aspiration to medicine as realistic ( Hill et al., 2004). Applicants are divided into school leavers and mature students and are tackled in different ways, as detailed below.

#### Outreach

All medical schools are required to provide outreach programmes ( BMA, 2010; MSC, 2014). These are activities of varying intensities aimed at ‘raising aspirations’ and addressing issues preventing disadvantaged students applying to medicine ( BMA, 2010). Low-intensity activities include open days or evenings, high-intensity projects include weekend programmes, mentoring and summer schools ( Brown & Garlick, 2006; Dismore, 2009). Activities often target students before they make subject choices which may prevent them accessing a medical education ( Brown & Garlick, 2006).

High-intensity activities often have a better impact than low-intensity ( Kamali et al., 2005). For example, mentoring improves success rates of disadvantaged applicants through supporting applications and providing practice interviews ( Kamali et al., 2005). However, students appear to prefer low-intensity activity, also favoured by institutions for being less costly ( Cleland et al., 2012).

#### Access to Medicine Courses

Access courses are another pre-medicine WP initiative, normally aimed at mature students. They differ from foundation courses, which are part of further education and therefore eligible for student finance and can lead directly into a medical degree ( Table 1) ( Parry et al., 2012). Access courses do not qualify for student finance, but may be free if the student is under 23-years old. Over 19-year olds may apply for a loan which is written off if a degree is completed ( SFA, 2016). These courses do not guarantee a route into medicine ( Holmes, 2002).

Applicants to access courses should not have the required academic qualifications to apply directly for medical school, this provides entry routes for mature students of non-traditional backgrounds. Often these groups are of lower SES ( Watson, 2011). WP, by supporting more diverse mature students, seems logical, but whilst numbers of graduate medical students have increased, there has not been an effect on socioeconomic diversity ( Mathers et al., 2011). This reflects the difficulty to adequately target disadvantaged mature applicants for access courses; for example, postcodes are often used, however these are not necessarily good indicators of a graduate applicants’ SES ( James et al., 2008).

**Table 1:** comparison of access and foundation courses ( Brown & Garlick, 2006; Holmes, 2002; Mathers et al., 2011; Parry et al., 2012; SFA, 2016)

	Access Course	Foundation Course
**Education level**	Higher education.	Further education.
**Entry requirements**	Applicants should not have the academic qualifications required to apply to medicine by traditional routes.	Applicants are generally required to have contextual data. The courses have lower academic entry requirements than traditional courses.
**Funding**	May be free if aged under 23, Advanced Learners Loan available for over 19-year olds or courses are eligible for Student Finance England.	Student Finance England.
**Length of study**	1 year.	6 years - 1 year of foundation course and then a standard medical degree.
**Route into Medicine**	Apply to standard entry medical school after finishing the access.	Progress straight into Year 1 of a standard medical degree.

### Application Process

Initiatives aimed at WP may be ‘too late’ when aimed at application processes, however ensuring WP applicants who do apply for medicine are not disadvantaged by the process is essential ( Brown & Garlick, 2006; O’Neill et al., 2013; Reiter et al., 2012). Determining selection tools which widen participation is also necessary if there is success in increasing the number of disadvantaged applicants to medical school ( Tiffin et al., 2012).

#### Contextual Data

Some medical schools are introducing contextual data (CD) to their admissions procedures. These are indicators used to identify candidates of lower SES, such as home postcode, school attended or if applicants are ‘first in family’ to attend university ( Cleland et al., 2014). They can then be used to identify and support medicine applicants in three main ways ( Cleland et al., 2014):

1. ‘Flagging’ - meeting a CD point
2. ‘Triangulation’ - meeting several CD points
3. ‘Flagging and adjusting’ - adjusting the requirements expected of identified students

Adjustments include offering lower academic entry requirements or highlighting identified applicants for ‘further consideration’ where they achieve borderline scores in admission processes. Their interviews may also be amended to focus less on work experience and extra-curricular activities ( Bridger et al., 2012).

Such steps aim to increase the success rates of identified CD applicants; however, CD indicators are new to admissions processes, therefore there is little evidence to support this and there remain considerable inconsistencies in the ways medical schools implement CD indicators ( Cleland et al., 2015). Many universities also do not offer reduced entry grades to CD applicants due to concerns of the impact this has on university league table rankings ( Moore et al., 2013).

Another issue with CD indicators is the difficulty experienced in identifying markers useful for all students. Triangulation is therefore recommended by the General Medical Council (GMC) in order to identify applicants who are truly disadvantaged ( Cleland et al., 2014).

#### Multiple Mini-Interviews and Situational Judgement Tests

Selection tools also have a role in WP to ensure disadvantaged students applying to medical school have a fair, equal chance of application success. Multiple mini-interviews (MMIs) and situational judgement tests (SJTs) are selection tools with the potential to increase diversity of successful applicants to medical school. The GMC’s ‘Selecting for Excellence’ Report determined that MMIs are capable of ‘moderately’ WP, whilst SJTs are capable of ‘moderate-highly’ doing the same ( Cleland et al., 2014; Selecting for Excellence Executive Group, 2014).

##### Multiple Mini-Interviews

There are many issues with traditional interviews as selection tools, particularly regarding reliability and validity ( Kreiter et al., 2004). MMIs are therefore being increasingly used as an improved way of interviewing medical school applicants ( Pau et al., 2013).

Research into the relationship between MMIs and WP is limited, however the evidence that does exist demonstrates no association between SES and MMI scores, although there are some issues relating to use among candidates with poorer English language proficiency ( Cleland et al., 2014; Kelly et al., 2014; Reiter et al., 2012). Larger scale studies are therefore needed before MMIs can be endorsed as being useful for WP, however these encouraging early results, combined with the ability to standardise questions and increase the candidate to interviewer ratio, make MMIs an attractive option for medical schools evaluating WP in their selection processes ( Rosenfeld et al., 2008).

##### Situational Judgement Tests

SJTs also have potential to widen participation and, as with MMIs, are found to be both reliable and valid ( Patterson et al., 2016). SJTs are designed to be ‘un-coachable’: this can be a major problem with other selection tools, as disadvantaged applicants may not have access to coaching and are therefore less able to compete ( Cullen et al., 2006). Again, there is limited research exploring the SJTs and WP, however they have been proven more resistant to coaching and produce scores unaffected by minority group status ( Christian et al., 2010; Cullen et al., 2006). SJTs have a further advantage in that they can be distributed electronically, meaning candidates do not have to travel to take the test. There is however little evidence supporting the predictive validity of SJTs on professional performance ( Cleland et al., 2012).

### Post Application

#### Foundation Courses

Foundation courses are 6-year medical courses, offered exclusively to students of lower SES ( Table 1). They have lower entry requirements and allow students to spend their first year of medicine developing knowledge and skills of benefit in HE ( Brown & Garlick, 2006; Mathers et al., 2011).

Foundation courses are perceived positively by students enrolled upon them and are linked with increased socioeconomic diversity in medical schools ( Garlick & Brown, 2008). This does vary, with newer medical schools’ foundation programmes generally better established and resulting in greater socioeconomic diversity ( Cleland et al., 2012). There is however a large investment of money and time required to run a foundation course; it is unclear whether the increased socioeconomic diversity of students gained is cost-effective ( Mathers et al., 2011).

An additional challenge for foundation courses, which may be relevant to other WP strategies, is the difficulty to determine whether students accessing the course are genuinely disadvantaged, or just traditional students who have previously missed out on medical education ( Searle, 2004). The use of CD indicators may help to reduce this possibility.

#### During Medical School

A final area recently highlighted as important regarding WP, is preventing the dropout of successful WP students after starting medical school. There are no data quantifying attrition rates of WP students, however they are perceived as more likely to fail and as ‘riskier’ students ( Cleland et al., 2012). They also need more ongoing support than other pupils, particularly as they may find it harder to form social identities with their medical student peers ( Brown & Garlick, 2006; Leathwood & O’Connell, 2003). Recent evidence however shows that students from state-funded schools outperform their peers who attended independent schools ( Kumwenda et al., 2017). The same finding may therefore also be true of WP candidates.

Medical schools are now encouraged to develop support mechanisms for disadvantaged applicants. Interventions designed to help students meet others, receive financial advice and develop subject-specific knowledge are best received ( Smith et al., 2012). When considering HE in general, disadvantaged students benefit from student-centred recognition of the social aspects of learning, support in the first year and early formative assessment ( Cleland et al., 2012). These measures are also likely to benefit disadvantaged medical students.

## Is WP really a good thing?

There are some arguments against WP, the particular concern for medical schools being that standards of achievement may drop ( Whiteford et al., 2013). Evidence demonstrates lower entry standards do not equate to lesser achievement in medical school, however WP students need support to achieve this, which poses an associated financial burden ( Brink, 2009; James et al., 2008; Taylor et al., 2009; Whiteford et al., 2013). Courses should be adjusted to support WP students, rather than providing interventions aimed at helping them adjust to the existing undergraduate ethos ( Whiteford et al., 2013).

There is also an argument that WP is not sufficient in itself to increase diversity in the medical profession. Prior to recent efforts to address socioeconomic inequality in medicine, considerable steps were taken to eradicate gender inequality ( Kilminster et al., 2007). This has generally been successful, with medical schools cohorts now over 50% female ( BMA, 2009). However, it has not fully translated into more female doctors in higher medical workforce roles. This ‘glass-ceiling’ effect is particularly prominent in some specialities, such as surgery ( Boursicot & Roberts, 2009; Loden, 1987). Thus, whilst equality may be established at medical school, social factors appear to prevent true gender diversification of the medical profession. There may be parallels here to SES and this raises concerns that WP does not address social factors’ impact on disadvantaged students throughout their careers.

## Conclusion

WP in medical education seems to be a necessity, underpinned by issues of social mobility and the understanding that a more diverse workforce will provide important healthcare benefits ( Girotti et al., 2015; Hayton & Paczuska, 2003; Magnus & Mick, 2000). Issues of WP have been recognised for two decades, with various steps being taken to reduce the socioeconomic inequality in HE and specifically in medical schools. However, little progress appears to have been made thus far ( BMA, 2009).

Medical schools currently adopt various strategies to widen participation, aimed at pre-, post- and intra-selection to institutions. Despite this, it must be recognised that there may be very little universities can do with certainty of success in individual measures. The inequalities to be addressed lie at the heart of our society, with those born into disadvantaged backgrounds already limited compared to their peers, through low motivation and cultural differences in educational attitudes ( Greenhalgh et al., 2004). The adoption of a ‘deficit’ model does not tackle the issues at their root and more needs to be done to address societal issues of inequality before WP is likely to be achievable ( Milburn et al., 2013).

## Take Home Messages

Medical schools can, and indeed are encouraged to, take steps to adopt a WP strategy in their own institution. Recommendations for these include:

• Offering high intensity outreach activities to the targets of WP.
• Considering disadvantage as well as academic grades when using an access course as a method of WP.
• Using CD to identify disadvantaged applicants during the medical school selection process and having a clear policy for how this CD will be used.
• Using MMIs in place of traditional interviews as a medical school selection tool.
• Remaining vigilant regarding the SJT literature and considering the possibility of introducing them to the medical school selection process.
• Encouraging foundation courses and using CD in the selection process.
• Supporting successful WP medical students, particularly during their early years at medical school.
• Considering how curricula can be adapted to meet the needs of such WP students.

## Notes On Contributors

Rosemarie Patterson is a fourth year medical student at Brighton and Sussex Medical School, Brighton, United Kingdom.

Dr Jim Price is the Programme Leader for Postgraduate Medical & Clinical Education at Brighton & Sussex Medical School (BSMS). He has been involved in the development of the Undergraduate Curriculum & Admissions procedures at BSMS since its inception in 2003 and strongly supports diversity and inclusive practice in all his educational programmes.
