# Supplementary material for: Widening participation in medicine: what, why and how?
Source: MedEdPublish (2016). 2017 Oct 13;6:184. [Version 1] doi: 10.15694/mep.2017.000184 (PMC10885306; doi:10.15694/mep.2017.000184)
Supplement: Peer review history

# Reviewer report: Reviewer response for version 1

**DOI:** 10.21956/mep.19370.r28355

**Published:** 2017-11-25

**Authors:** Barbara Jennings

**Affiliations:** 1 Norwich Medical School

This review has been migrated. The reviewer awarded 5 stars out of 5 This is an excellent summary of the WP challenges in medicine, with a thorough description of the range of interventions that are in use and an exploration of ethos and underlying assumptions.Given the current SES profile of students in UK medical school, it is clear that evidence-based interventions are essential to achieve equity of access to medical careers. This in turn is essential, because we need to recruit more doctors and can't risk selection from an unnecessarily narrow field.One data set I would like to see is the distribution of science A level results with respect to polar data, do the authors know where I could find this? I think it would be useful for considering adjusted entry tariffs based on contextual criteria.

Reviewer Expertise:

NA

No decision status is available

# Reviewer report: Reviewer response for version 1

**DOI:** 10.21956/mep.19370.r28353

**Published:** 2017-10-19

**Authors:** Ronald M Harden

**Affiliations:** 1 AMEE

This review has been migrated. The reviewer awarded 5 stars out of 5 Widening participation to medical studies is certainly on today's agenda in medical education. This well written and thoughtful article raises from a theoretical and practical perspective key issues that should be considered. The problems identified should not be seen in isolation . There is a need as suggested to look at the implications for the approach during medical school. This can best be seen as part of an adaptive curriculum where the programme is tailored to the needs of each individual student.

Reviewer Expertise:

NA

No decision status is available

# Reviewer report: Reviewer response for version 1

**DOI:** 10.21956/mep.19370.r28350

**Published:** 2017-10-15

**Authors:** Trevor Gibbs

**Affiliations:** 1 AMEE

This review has been migrated. The reviewer awarded 5 stars out of 5 A very well written and laid out paper that covers a very important area. As someone who came from a very low SES section of the community it particularly resonates with me and why I feel that I chose general practice and later on, international medical education as my two main careers.The paper is well researched with a very helpful set of references for anyone investigating the subject of widening access. It can be said that it is UK-centric, but we learn from each other and there are plenty of ideas that can be transferred to others dealing with the same or similar issues.I would certainly recommend this paper to anyone involved with selection to both medical school and other health professional schools.

Reviewer Expertise:

NA

No decision status is available

# Reviewer report: Reviewer response for version 1

**DOI:** 10.21956/mep.19370.r28354

**Published:** 2017-10-15

**Authors:** Paolo Colet

**Affiliations:** 1 Nazarbayev University

This review has been migrated. The reviewer awarded 5 stars out of 5 This is an interesting review article, although, it focuses on the medical schools only in the UK. Personally, I find it informative as it discusses political theory and recruitment strategies used by several medical schools to advance Widening Participation. The take-home messages, figure, and table presentation are rightfully included as they make easier to grasp the context of the review. There are few of the strategies presented that we are currently using at our medical school, and we still have much work to do. The review article deserves to be read by educators not only in medicine but also in other health-related programs from developed and developing countries.

Reviewer Expertise:

NA

No decision status is available

# Reviewer report: Reviewer response for version 1

**DOI:** 10.21956/mep.19370.r28352

**Published:** 2017-10-14

**Authors:** Neel Sharma

**Affiliations:** 1 Albert Einstein College of Medicine, Montefiore Medical Center

This review has been migrated. The reviewer awarded 5 stars out of 5 A fantastic review of widening participation. I certainly agree with the issues of elitism in medicine. The north south divide within the UK for example is still ever present within medical education. However this will ultimately have to change in view of changing economies if the UK is keen to remain a global player.

Reviewer Expertise:

NA

No decision status is available

# Reviewer report: Reviewer response for version 1

**DOI:** 10.21956/mep.19370.r28351

**Published:** 2017-10-13

**Authors:** John Cookson

**Affiliations:** 1 University of Worcester

This review has been migrated. The reviewer awarded 5 stars out of 5 Thank youI found this a very useful account of the situation. Although it has a UK focus, it will be valuable to others. UK educators will find it helpful in meeting the goverment's agenda for a more socially inclusive medical school intake

Reviewer Expertise:

NA

No decision status is available
